# Protease-Activated Receptor Type 1 Activation Enhances Osteogenic Activity in Human Periodontal Ligament Stem Cells

**DOI:** 10.1155/2019/6857386

**Published:** 2019-06-02

**Authors:** Emanuel Silva Rovai, Lucas Macedo Batitucci Ambrósio, Bruno Nunes de França, Letícia Rodrigues de Oliveira, Letícia Miquelitto Gasparoni, Carla Renata Sipert, Marinella Holzhausen

**Affiliations:** ^1^Division of Periodontics, Department of Stomatology, School of Dentistry, University of São Paulo, São Paulo, Brazil; ^2^Division of Endodontics, Department of Restorative Dentistry, School of Dentistry, University of São Paulo, São Paulo, Brazil

## Abstract

Protease-activated receptor 1 (PAR_1_) has been associated to tissue repair and bone healing. The aim of the present study was to evaluate the effect of PAR_1_ activation on the osteogenic activity of human periodontal ligament stem cells (PDLSCs). PDLSCs were cultured in the presence of PAR_1_-selective agonist peptide (100 nM), thrombin (0.1 U/mL), or PAR_1_ antagonist peptide (100 nM). Calcium deposits, calcium concentration (supernatant), alkaline phosphatase activity (ALP), cell proliferation, and gene (qPCR) and protein expression (ELISA assay) of osteogenic factors were assessed at 2, 7, and 14 days. PAR_1_ activation led to increased calcium deposits (*p* < 0.05), calcium concentration (*p* < 0.05), ALP activity (*p* < 0.05), and cell proliferation (*p* < 0.05). Further, PAR_1_ activation may increase gene and protein expression of Runx2 (*p* < 0.05) and OPG (*p* < 0.05). In conclusion, PAR_1_ activation increases osteogenic activity of PDLSCs, providing a possible new strategy for periodontal regenerative therapies.

## 1. Introduction

Periodontitis is an inflammation of the periodontal tissues which results in the loss of alveolar bone and tissue attachment surrounding the teeth [1]. The regeneration of periodontal tissues lost as a consequence of periodontal disease corresponds to the main goal of periodontal therapy [2]. Since it was demonstrated that stem cells have the capacity to differentiate and expand into different cell lineages maintaining the specific functions, its use becomes an interesting therapeutic option [3]. Periodontal ligament stem cells (PDLSCs) are able to differentiate into osteoblasts, cementoblasts, and fibroblasts and play an important role in the regeneration of periodontal tissues [4]. Further, Seo et al. [5] showed that when transplanted into periodontal defects surgically created in mice, PDLSCs can lead to periodontal ligament regeneration and may be associated with the trabecular bone regenerated in the periodontium, therefore suggesting a potential role of PDLSCs in the regeneration of bone tissue.

PAR_1_ was the first cloned member of the G protein-coupled receptor family. The proteolytic cleavage of PAR_1_ determines a new N-terminal sequence which binds to the receptor itself, resulting in its automatic activation, generating an intracellular signaling pattern [6]. PAR_1_ is expressed by several periodontal cell types, such as gingival epithelial cells [7], human gingival fibroblasts [8], osteoblasts [9], periodontal ligament cells [10], and monocytic cells [11], and its endogenous activators, such as thrombin, plasmin, and matrix metalloproteinases (MMPs), are present in the periodontium.

PAR_1_ plays an important role in periodontal tissue metabolism [12], since its activation has been associated to fibroblast proliferation [13], and release of connective tissue growth factor (CTGF) and transforming growth factor beta (TGF-*β*) [14]. In addition, da Silva et al. [15] demonstrated that PAR_1_ was overexpressed by epithelial and immune cells from the gingival crevicular fluid after periodontal treatment, therefore suggesting its possible protective role during periodontal repair in patients with chronic periodontitis.

Interestingly, it was shown that PAR_1_ activation regulates several aspects of osteoblast function and bone repair [16, 17], such as osteoblast proliferation [18], and expression of TGF-*β*, fibroblast growth factor type 1 (FGF-1) and type 2 (FGF-2), and CTGF [9, 19]. In addition, Arayatrakoollikit et al. [10] demonstrated in periodontal ligament cells that PAR_1_ activation could increase the synthesis of osteoprotegerin (OPG), a protein that regulates bone homeostasis and osteoclast activation. Corroborating with these findings, a recent study [20] observed that a plasminogen-activating protease can reduce the inflammatory osteoclastogenesis induced by LPS through PAR_1_ activation. Furthermore, in an animal model using PAR_1_-null mice, it was shown that PAR_1_ acts on the proliferation of bone marrow stromal cells and is related to increased bone formation and fewer osteoclasts, playing an important role in the early stages of bone healing [19].

Taken together, these findings suggest that PAR_1_ may play a role in the regeneration of periodontal tissues. The hypothesis of the present study is that PAR_1_ is associated to increased osteogenesis in human PDLSCs. Thus, the aim of the present study was to evaluate the effect of PAR_1_ activation on the osteogenic activity in PDLSCs.

## 2. Material and Methods

### 2.1. Isolation and Characterization of the Periodontal Ligament Stem Cells

Human periodontal ligament cells were collected from healthy third molars from three different patients at the clinic of the School of Dentistry of the University of São Paulo (FO-USP). All individuals who agreed to participate signed a term of free informed consent. This study was approved by the Ethics Committee of the FO-USP Research Ethics Committee under the protocol # 803.811.

Periodontal ligament tissue was taken from the middle third of the root, and cell culture was established using the explant technique [21]. Cells were cultured in control medium (CM) composed of alpha-modified Eagle's medium (*α*-MEM) supplemented with 15% fetal bovine serum, 100 *μ*g/mL penicillin, 100 *μ*g/mL streptomycin, and 0.5 mg/mL amphotericin B (all from Gibco, Invitrogen, Carlsbad, CA, USA) and incubated at 37°C with 5% CO_2_ and 95% air humidity. Once confluent, cells were trypsinized and subcultured. Cells from 3 different subjects were used for experiments at passages 3-7.

Flow cytometry was used in order to examine cell surface markers. Approximately 5 × 10^5^ cells were isolated, washed in phosphate buffered saline (PBS), and incubated for 30 min at 4°C with the following monoclonal antibodies (eBioscience, San Diego, USA): CD14-FITC, CD90-FITC, CD34-FITC, CD31-PE, CD44-PE, CD45-PE, and CD146-PE. Cell suspension was washed twice with PBS and analyzed with the FACSort flow cytometer (Becton Dickinson, Brazil). The recorded events were analyzed using the CellQuest software (Becton Dickinson, Brazil).

### 2.2. Experimental Groups

Human PDLSCs were seeded in 24-well plates at a density of 25000 cells/cm^2^ and cultured with CM or osteogenic medium (OM) (CM + 0.1 mM dexamethasone, 2 mM *β*-glycerophosphate, and 50 *μ*g/mL ascorbic acid; all from Sigma-Aldrich, St. Louis, MO, USA), and experimental groups were treated with PAR_1_-selective agonist peptide TFLLR-NH2 (100 nM) [22] (Tocris Bioscience Inc., Bristol, UK) or thrombin (0.1 U/mL) (Sigma-Aldrich, St. Louis, MO, USA) for distinct experimental periods based on the analysis performed. To confirm whether the thrombin-induced effect was specifically mediated by PAR_1_, cultures were pretreated with PAR_1_-selective antagonist RWJ 56110 (100 nM) [23] (Tocris Bioscience Inc., Bristol, UK) for 30 min prior to thrombin stimulation. Culture medium with its specific treatments (PAR_1_ agonist, thrombin, and thrombin + PAR_1_ antagonist) was changed every two days. Supernatant and cells were collected for further analysis.

### 2.3. Mineralized Nodule Formation (Alizarin Red Staining)

In vitro mineralization was evaluated at 7 and 14 days by alizarin red staining (Sigma-Aldrich, St. Louis, MO, USA). Briefly, cells were washed with cold PBS and then fixed in 10% formaldehyde for 30 min at room temperature. Cultures were then washed twice with distilled water and exposed to 1 mL 40 mM alizarin red solution (pH 4.1) per well for 30 min at room temperature. After staining, cells were washed with distilled water and digital images of the mineral deposits were visualized using an inverted microscope (TMS 211124, Nikon, Japan). Quantification of mineralized nodule formation was assessed as previously described by Gregory et al. [24], with aliquots (150 mL) of the supernatant read at 405 nm and 550 nm in 96-well format using a spectrophotometer (BioTek Instruments, Winooski, VT, USA). All reactions were made in duplicate.

### 2.4. Calcium Concentration and Alkaline Phosphatase (ALP) Activity

ALP activity and calcium concentration in the culture medium (supernatant) were assessed at 2, 7, and 14 days. Samples were measured by using a commercial colorimetric kit (Abcam, Cambridge, MA, USA), according to the manufacturer's instructions. All reactions were made in duplicate.

### 2.5. Cell Proliferation

Cell proliferation was measured at 2 and 4 days in CM and OM after each of the 4 treatments proposed (control, PAR_1_ agonist, thrombin, and PAR_1_antagonist + thrombin) using the Quick Cell Proliferation Assay Kit (Abcam, Cambridge, MA, USA), according to the manufacturer's instructions. All reactions were made in duplicate.

### 2.6. Cell Expression of Osteogenic Genes

Gene expression of Runx2, OPG, receptor activator of nuclear factor kappa-B ligand (RANKL), and osteocalcin (OC) was evaluated by reverse transcription followed by quantitative PCR (RT-qPCR) in samples collected at 2, 7, and 14 days in CM and OM after each of the 4 treatments proposed (control, PAR_1_ agonist, thrombin, and PAR_1_antagonist + thrombin).

Total RNA was extracted in 1 mL TRIzol reagent (Invitrogen, Carlsbad, CA, USA) per well. Through a reverse transcription reaction, complementary DNA (cDNA) was synthesized using the High Capacity RNA-to-cDNA Kit (Applied Biosystems, Foster City, CA, USA) and RT-qPCR was performed using TaqMan Universal Master Mix II (Applied Biosystems, Foster City, CA, USA). The standard PCR conditions were 95°C (10 min) and then 40 cycles of 95°C (15 sec), 60°C (1 min) and a final cycle with an increasing temperature from 60°C to 95°C (20 min) to obtain a standard denaturation curve. GeneBank accession numbers of the oligonucleotide sequences used for cDNA amplification were as follows: OPG (Hs00171068-m1), Runx2 (NM-004348), RANKL (Hs00243519-m1), OC (Hs00609452_g1), and GAPDH (NM_002046). The relative levels of gene expression were calculated based on the reference sample (untreated control) normalized to the housekeeping gene (GAPDH). Samples without RNA and without reverse transcriptase were used as negative controls. All reactions were made in duplicate.

### 2.7. Osteogenic-Related Protein Expression

In culture medium samples (supernatant), levels of Runx2, OPG, OC, and osteopontin (OPN) were assessed by the use of commercially available enzyme-linked immunosorbent assay (ELISA) kits (MyBioSource.com, San Diego, CA, USA), according to the manufacturer's instructions. Reactions were made in triplicate and the results were expressed in pg/mL.

### 2.8. Statistical Analysis

Statistical analysis of the results was carried out with the aid of the program GraphPad Prism 5.01 (GraphPad Software, La Jolla, CA, USA). All data obtained were representative of three independent experiments performed with cells derived from three different donors. All analyzes were performed with a significance level of 5%. ANOVA test was used for parametric data and the Kruskal Wallis test for all non-parametric analyzes.

## 3. Results

### 3.1. Cell Characterization

**Figure 1:**
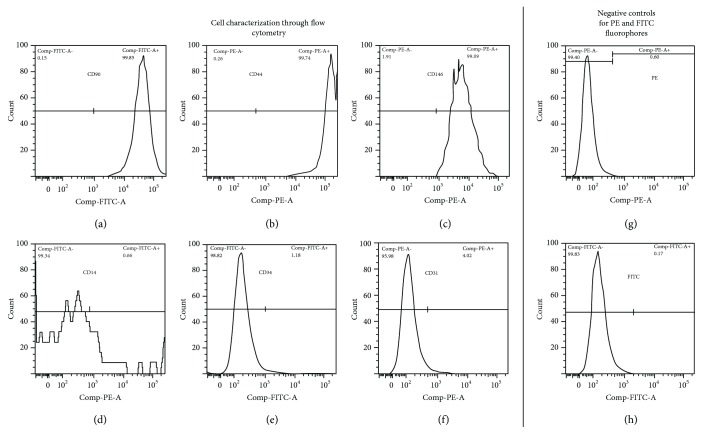
Cell characterization through flow cytometry.

Flow cytometry was used in order to examine cell surface markers (Figure 1). Cells were positive for CD146, CD44, and CD90. On the other hand, cells were negative to CD14, CD34, and CD31.

### 3.2. PAR_1_ Activation Increased Mineralized Nodule Formation

**Figure 2:**
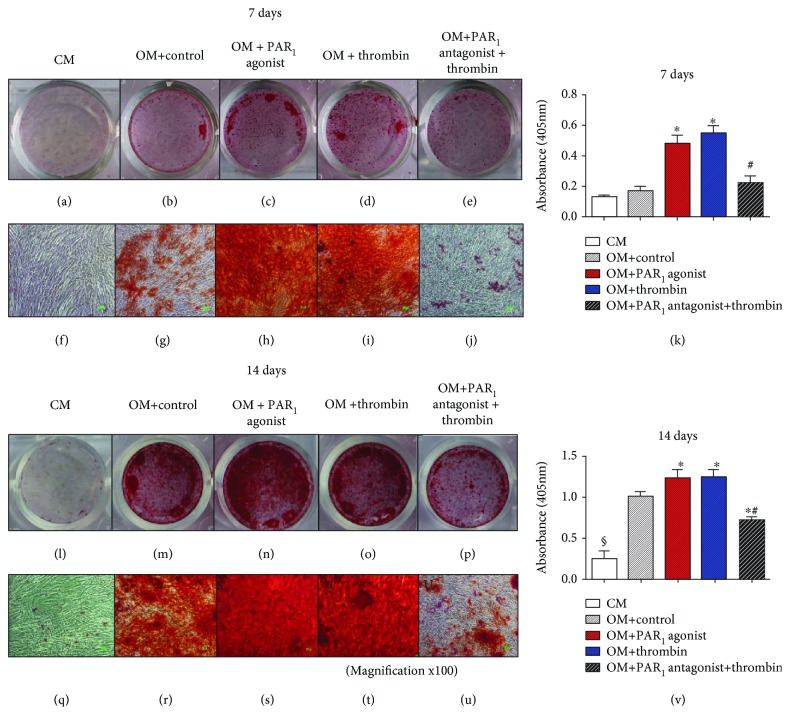
(a–j and l–u) Effects of PAR_1_ on mineral deposition using alizarin red staining after 7 and 14 days (a–e and l–p: zero magnification; f–j and q–u: magnification ×100). (k, v) Quantitative alizarin red staining analysis after 7 and 14 days. Data are in mean and SD; *n* = 3. ^∗^Mean significant difference when compared to OM + control (*p* < 0.05). ^#^Mean significant difference when compared to OM + thrombin (*p* < 0.05). ^§^Mean significant difference when compared to OM groups (*p* < 0.05).

Mineralized nodule formation was assessed with alizarin red staining at 7 and 14 days (Figure 2). After 7 days, both PAR_1_ agonist peptide and thrombin treatment led to significantly increased mineralized nodule formation compared to controls (*p* < 0.05). At 14 days, all groups, except CM, were positive for alizarin red staining. However, PAR_1_ activation by its synthetic agonist peptide or thrombin resulted in significantly increased mineralized nodule formation compared to controls (*p* < 0.05).

In addition, treatment with PAR_1_-selective antagonist peptide abolished the thrombin-positive effect on mineralized nodule formation at 7 and 14 days, therefore suggesting that its action was specifically mediated by PAR_1_ (Figures 2(a)–2(v)).

### 3.3. Effect of PAR_1_ Activation on ALP Activity and Calcium Concentration

**Figure 3:**
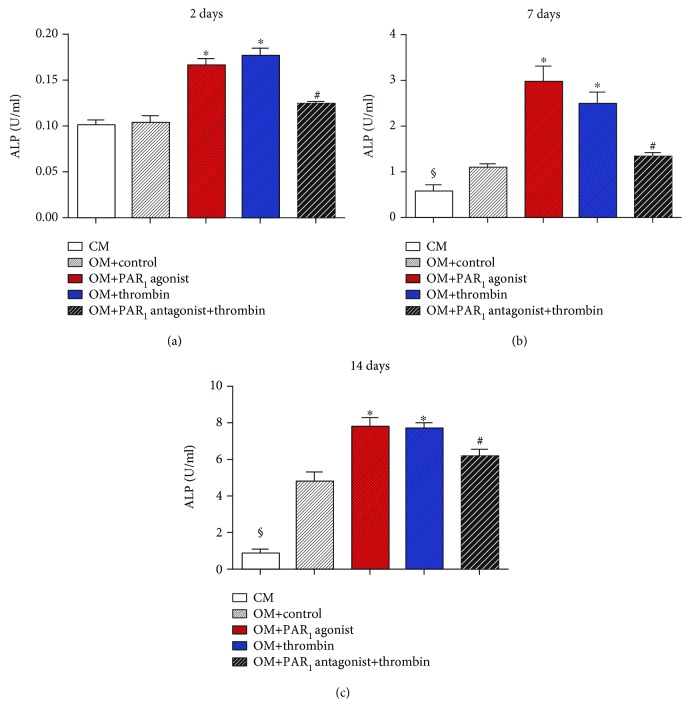
ALP activity in different groups. Values are presented as mean and SEM; *n* = 3. ^∗^Mean significant difference when compared to OM + control (*p* < 0.05). ^#^Mean significant difference when compared to OM + thrombin (*p* < 0.05). ^§^Mean significant difference when compared to OM groups (*p* < 0.05).

ALP activity was assessed at 2, 7, and 14 days (Figure 3). In all experimental periods, PAR_1_ agonist peptide and thrombin resulted in a stronger ALP activity compared to control groups (*p* < 0.05). In addition, thrombin-induced ALP activity was significantly decreased after PAR_1_ antagonist peptide treatment (*p* < 0, 05).

**Figure 4:**
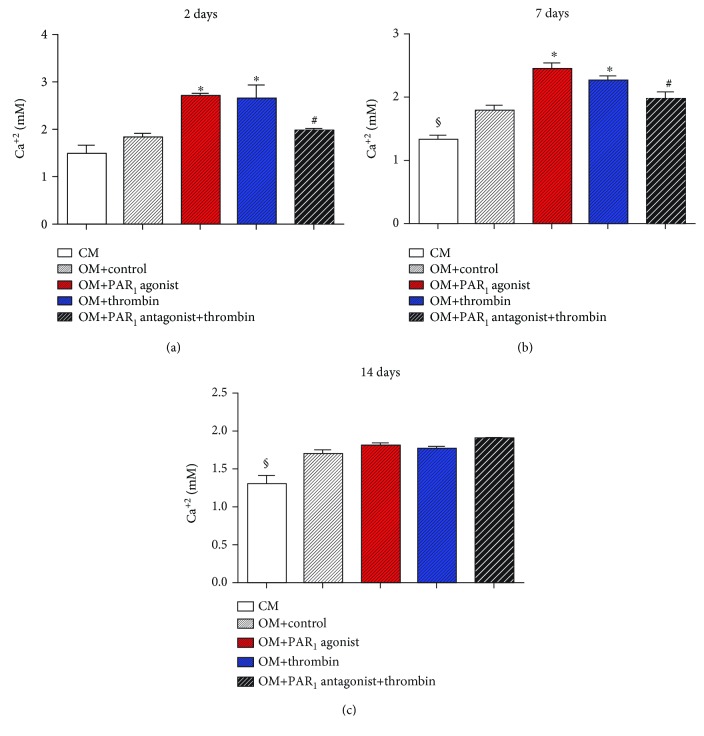
Calcium concentration in the supernatant in different groups. Values are presented as mean and SEM; *n* = 3. ^∗^Mean significant difference when compared to OM + control (*p* < 0.05). ^#^Mean significant difference when compared to OM + thrombin (*p* < 0.05). ^§^Mean significant difference when compared to OM groups (*p* < 0.05).

Calcium concentration was assessed at 2, 7, and 14 days of experiment (Figure 4). At 2 and 7 days, PAR_1_ agonist peptide and thrombin treatments led to significantly increased calcium concentration in comparison with control groups (*p* < 0.05). Moreover, thrombin-induced calcium concentration was significantly decreased after PAR_1_ antagonist peptide treatment (*p* < 0.05). At 14 days, no significant effect of PAR_1_ activation on calcium concentration was observed in groups treated with osteogenic medium.

### 3.4. PAR_1_ Activation Increased Cell Proliferation

**Figure 5:**
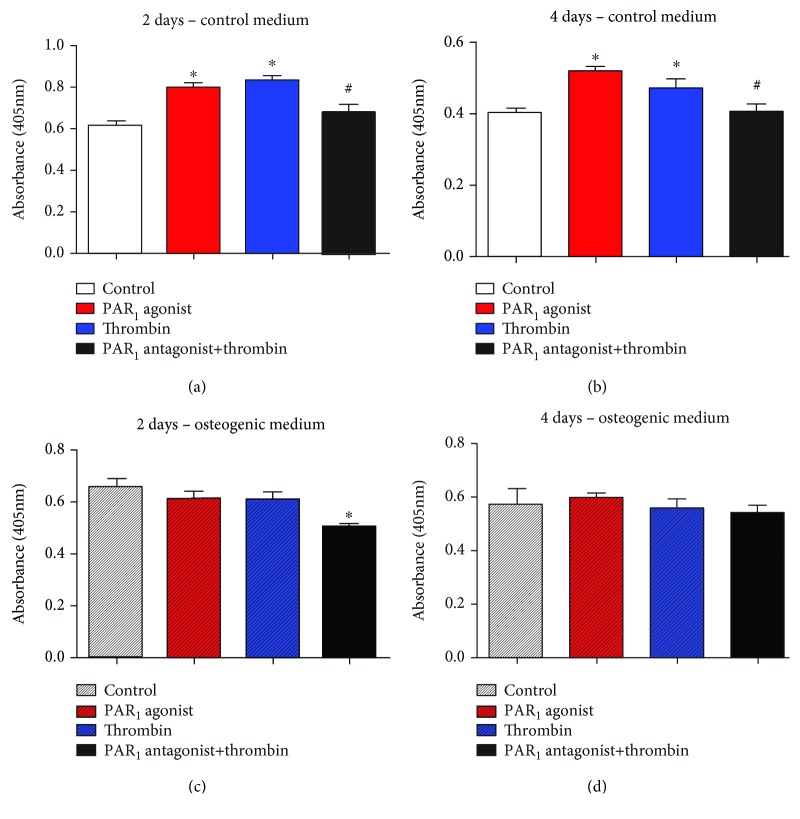
Cell proliferation in different groups. Values are presented as mean and SEM; *n* = 3. ^∗^Mean significant difference when compared to control of the same medium and time point (*p* < 0.05). ^#^Mean significant difference when compared to thrombin treatment of the same medium and time point (*p* < 0.05).

Cell proliferation was assessed at 2 and 4 days (Figure 5). PAR_1_ activation through PAR_1_ agonist peptide or thrombin led to increased cell proliferation in the control medium. In addition, PAR_1_-selective antagonist significantly decreased cell proliferation by thrombin at 2 days in both control and osteogenic medium (*p* < 0.05). No significant difference was found among groups at 4 days in the osteogenic medium.

### 3.5. Gene and Protein Expression

**Figure 6:**
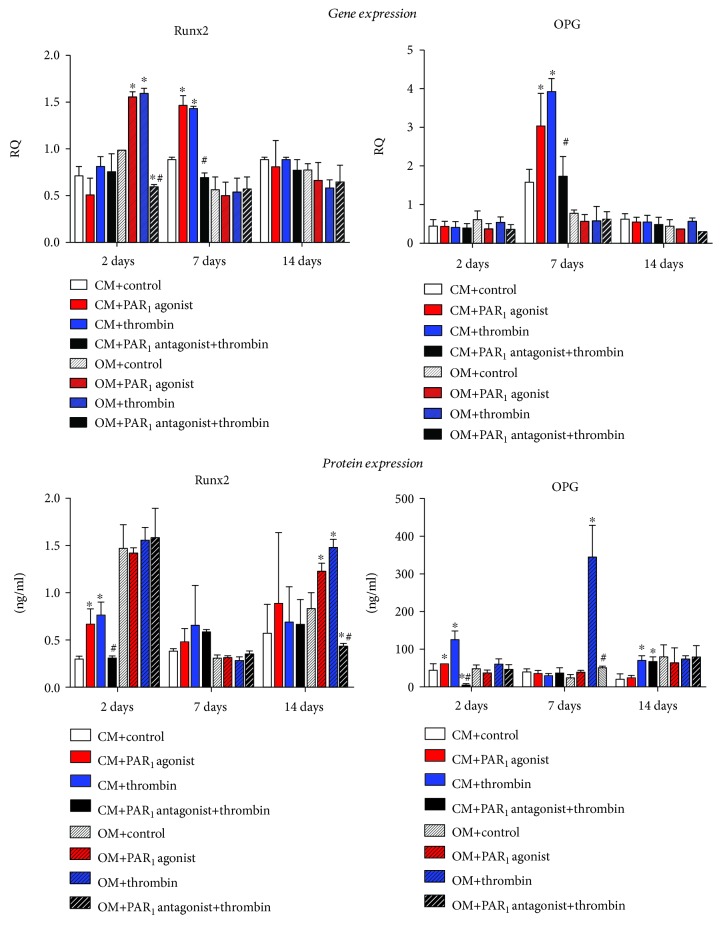
Effects of PAR_1_ activation on Runx2 and OPG protein and gene expression at 2, 7, and 14 days. Values are presented as mean and SEM; *n* = 3. ^∗^Mean significant difference when compared to control of the same medium and time point (*p* < 0.05). ^#^Mean significant difference when compared to thrombin treatment of the same medium and time point (*p* < 0.05).

In order to clarify the mechanisms involved in mineralization, osteogenic gene expression (Runx2, OPG, OC, and RANKL), and protein expression (Runx2, OPG, OPN, and OC) were assessed by real-time PCR and ELISA, respectively. In these experiments, cells were treated with CM and OM in the presence of PAR_1_-selective agonist peptide, thrombin, or PAR_1_ antagonist peptide + thrombin (Figure 6).

At 2 days, treatment with both PAR_1_ agonist peptide and thrombin stimulated Runx2 gene expression in the osteogenic medium and protein expression in the control medium (*p* < 0.05). At 7 days, both PAR_1_ agonist and thrombin treatments increased Runx2 gene expression in control medium (*p* < 0.05). At 14 days, PAR_1_ activation by PAR_1_ agonist or thrombin treatments increased Runx2 protein expression in the osteogenic medium (*p* < 0.05). In addition, PAR_1_ blockade with its selective antagonist prevented both gene- and protein-increased expression of Runx2 by thrombin.

OPG protein expression at 2 days and gene expression at 7 days were elevated after PAR_1_ activation by PAR_1_ agonist or thrombin in comparison with control (*p* < 0.05) in the control medium, whereas treatment with PAR_1_ antagonist prevented this effect (*p* < 0.05). At 7 days in the osteogenic medium, thrombin profoundly increased OPG protein expression compared to all groups (*p* < 0.05), whilst PAR_1_ blockade with its specific antagonist significantly decreased its expression. Further, after 14 days, treatment with thrombin elevated OPG protein expression in comparison with control and PAR_1_-selective agonist peptide groups (*p* < 0.05) in the control medium. Interestingly, PAR_1_ blockade with its selective antagonist did not prevent protein-increased expression of OPG by thrombin.

**Figure 7:**
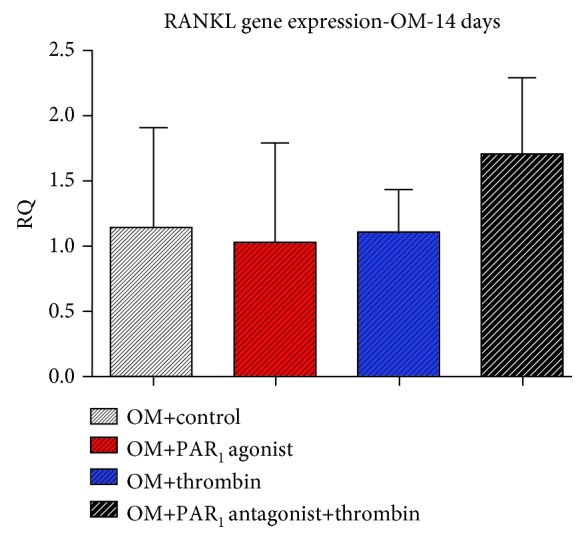
Effects of PAR_1_ activation on RANKL gene expression at 14 days, in the osteogenic medium. Values are presented as mean and SEM; *n* = 3.

**Figure 8:**
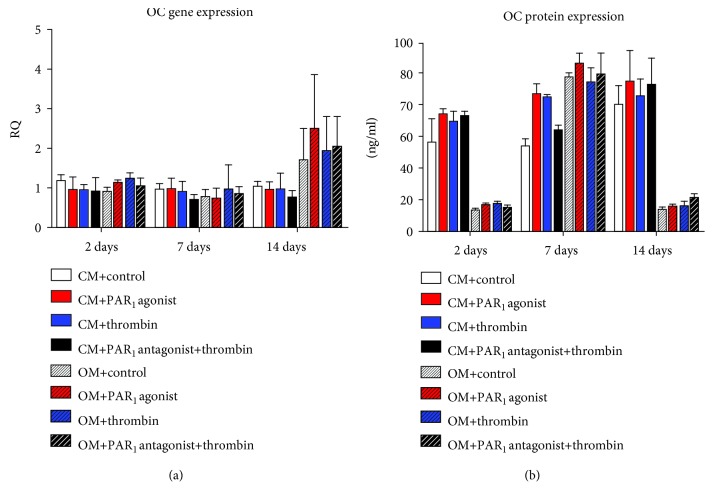
Effects of PAR_1_ activation on OC protein and gene expression at 2, 7, and 14 days. Values are presented as mean and SEM; *n* = 3.

**Figure 9:**
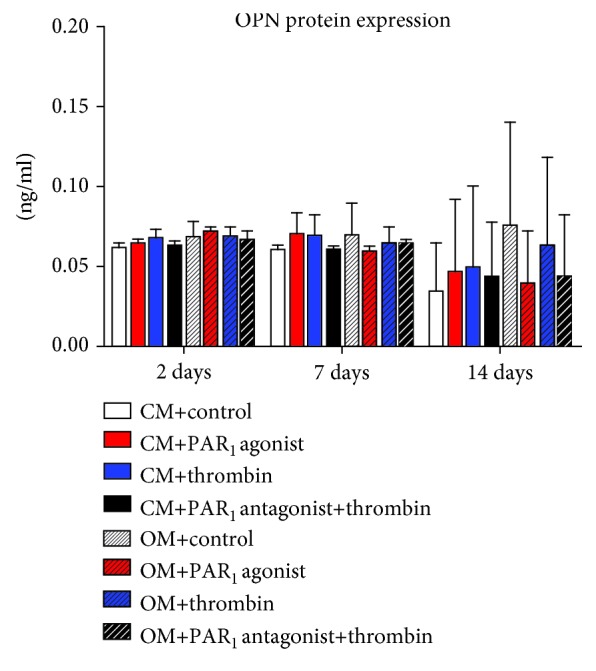
Effects of PAR_1_ activation on OPN protein expression at 2, 7, and 14 days. Values are presented as mean and SEM; *n* = 3.

There were no significant differences among treatments in any time point and culture medium regarding RANKL, OC, and OPN gene and protein expression (Figures 7–9, respectively).

## 4. Discussion

The main results presented herein indicate for the first time that PAR_1_ activation may enhance the osteogenic activity in human PDLSCs by increasing the formation of calcium deposits, ALP activity, PDLSC proliferation, and expression of osteogenic factors.

Initially, cells obtained from the periodontal ligament of three donors were characterized by flow cytometry. The results demonstrated that the cells were positive for CD146, CD90, and CD44 and at the same time negative for CD14, CD31, and CD34, being that these data are in agreement with the literature for the characterization of PDLSCs [25].

PAR_1_ activation in PDLSCs by its selective agonist peptide or by thrombin resulted in increased mineralized nodule formation and calcium concentration, thus suggesting that PAR_1_ plays a pivotal role in the mineralization process. Corroborating with these findings, the literature shows an important role of PAR_1_ in bone metabolism and healing [9, 16, 17, 19].

One could speculate that thrombin-induced mineralization was not specifically mediated by the activation of PAR_1_, since it is known that thrombin can also activate PAR_3_ and PAR_4_ [6]. However, PAR_1_ blockade before thrombin activation decreased the formation of calcium deposits, therefore indicating that thrombin-derived increased calcium deposits were specifically mediated by the activation of PAR_1_. It is believed that plasmin, thrombin, and MMPs, some possible endogenous PAR_1_ activators, may be present during bone formation and repair [16]. It was previously shown by da Silva et al. [15] that both PAR_1_ and MMP-13 are increased after periodontal treatment. Interestingly, MMP-13 has been shown to be expressed by human mesenchymal cells during osteogenic differentiation playing an important role in osteoblastic differentiation as well as alveolar bone formation and repair [26]. In our study, PAR_1_ blockade by its selective antagonist peptide not only has shown that thrombin-induced mineralization was specifically mediated by PAR_1_ but also suggested that an endogenous PAR_1_ activator, possibly MMP-13, was present at the osteogenic medium, since it was demonstrated that the antagonist peptide resulted in significantly less alizarin red staining compared to the control at 14 days (*p* < 0, 05). Therefore, our results clearly demonstrate a determinant role of PAR_1_ in mineralization and differentiation of PDLSCs.

Noteworthy, since the culture media was changed every 28 hours, the source of calcium ions in the culture media may have influenced the results. Moreover, increased osteogenesis is expected to increase mineral deposition, in particular calcium and phosphate into the extracellular matrix, as indicated in Figure 2, which would first increase calcium concentration in the supernatant and then decrease as the calcium deposition process takes place. These abovementioned facts can explain why PAR1 activation lead to increases in calcium concentration only at the initial time points of 2 and 7 days.

PAR_1_ activation, by its selective agonist peptide or thrombin, resulted in the significantly increased activity of ALP, an important early marker of osteoblast differentiation [27]. In addition, it was shown that thrombin-derived increased activity of ALP is specifically mediated by PAR_1_, since the addition of the antagonist peptide significantly decreased its effect. Corroborating with these findings, in primary rat osteoblast-like cells, Abraham and Mackie [18] found in a subset analysis of different cell populations that in immature cells, the treatments with thrombin and PAR_1_-activating peptide increased ALP activity. A possibility raised by these authors was that thrombin via PAR_1_ activation may directly stimulate differentiation of osteoblast precursor cells. In addition, another possibility raised by these authors was that PAR_1_ activation may enhance the proliferation of a subset of cells that exhibit a more osteoblast-like phenotype generating a larger number of cells for the differentiation process. On the other hand, PAR_1_ activation has also been implicated in ALP activity inhibition in more mature preosteoblasts and the main reason is that in this type of cells, PAR_1_ activation stimulates proliferation [18, 28] and during this process, osteoblast differentiation is downregulated [29] hence resulting in the reduction of ALP activity.

It is known that thrombin exerts a mitogenic action [29, 30]. In fact, the thrombin proliferative potential via PAR_1_ signaling has been shown in osteoblasts [18, 28], bone marrow stromal cells [19], fibroblasts [14], chondrocytes [31], and astrocytes [32]. In the present study, thrombin increased PDLSC proliferation compared to control specifically via PAR_1_ activation. Interestingly, PAR_1_ activation by thrombin or its selective agonist peptide had no proliferative effect on PDLSCs in the osteogenic medium. This result may be explained by the fact that cell proliferation is inhibited during the cell differentiation process [18] which is stimulated by the osteogenic supplements of the medium.

Runx2 is a member of the runt domain family of transcription factors and regulates various aspects of osteoblast differentiation [33]. Levels of Runx2 are gradually increased during osteoblast differentiation, and inhibition of Runx2 blocks the differentiation of mesenchymal cells to osteoblasts [34, 35]. It can be suggested that the osteogenic differentiation outcomes followed by PAR_1_ activation are mediated by Runx2, since both PAR_1_ agonist peptide and thrombin led to significantly increased Runx2 expression in PDLSCs. It is known that Runx2 expression varies according to the regulation of the osteogenic microenvironment during the osteogenic differentiation process [36]. This fact could explain the fact that significant differences among groups were found only in some time points in the present study. One could hypothesize from our data that PAR_1_ activation may have highlighted the peaks of Runx2 expression during the osteogenic differentiation process. Further, although increased Runx2 protein expression at 2 and 7 days was not observed, a not assessed increase during the mean time of 2 and 7 days mayhave occurred, especially because it was shown an increased Runx2 gene expression at 2 days.

In the present study, PAR_1_ activation significantly increased OPG expression in PDLSCs at 2 days of experiment. These data suggest that the PAR_1_-induced synthesis of OPG in PDLSCs could also explain the proosteogenic effects that result from the activation of the receptor. Furthermore, since PAR_1_ activation increased OPG expression but had no effect on RANKL expression, these findings indicate that PAR_1_ can increase the proportion of OPG to RANKL, hence inhibiting osteoclastogenesis. Similar findings were described by Arayatrakoollikit et al. [10] that showed that PAR_1_ activation in periodontal ligament cells could increase the proportion of OPG to RANKL. Interestingly, at 7 days, thrombin profoundly induced OPG synthesis, whereas treatment with PAR_1_ agonist peptide had no effect on OPG expression. In addition, at 14 days, both thrombin and PAR_1_ antagonist treatments increased OPG protein expression, therefore suggesting that in the later stages of our experiment, OPG expression in PDLSCs was not mediated by the activation of PAR_1_.

Furthermore, PAR_1_ activation had no effect on OC and OPN expression. It is known that the levels of OC and OPN are enhanced at the later stages of bone formation and remodeling [37]. This is an additional evidence pointing to the more relevant effect of PAR_1_ activation at the early stages of osteogenesis playing determinant roles on mesenchymal cell proliferation and osteoblast differentiation in PDLSC culture.

## 5. Conclusions

In conclusion, the present study clearly demonstrates that PAR_1_ activation results in increased osteogenic activity in PDLSCs associated with an ultimately enhanced mineralized nodule formation as a consequence of its pivotal effects on cell proliferation and osteoblast differentiation, probably mediated by Runx2 and OPG. These findings suggest that PAR_1_ activation in PSLSCs may have a possible potential to enhance hard tissue regeneration of the periodontium.

## Data Availability

All data used to support the findings of this study are included within the article.

## References

[1] American Academy of Periodontology (2001). *Glossary of Periodontal Terms*.

[2] Wu Y.-C., Lin L.-K., Song C.-J., Su Y.-X., Tu Y.-K. (2017). Comparisons of periodontal regenerative therapies: a meta-analysis on the long-term efficacy. *Journal of Clinical Periodontology*.

[3] Hu L., Liu Y., Wang S. (2018). Stem cell-based tooth and periodontal regeneration. *Oral Diseases*.

[4] Sowmya S., Chennazhi K. P., Arzate H., Jayachandran P., Nair S. V., Jayakumar R. (2015). Periodontal specific differentiation of dental follicle stem cells into osteoblast, fibroblast, and cementoblast. *Tissue Engineering Part C: Methods*.

[5] Seo B.-M., Miura M., Gronthos S. (2004). Investigation of multipotent postnatal stem cells from human periodontal ligament. *The Lancet*.

[6] Ramachandran R., Altier C., Oikonomopoulou K., Hollenberg M. D. (2016). Proteinases, their extracellular targets, and inflammatory signaling. *Pharmacological Reviews*.

[7] Rohani M. G., Beyer R. P., Hacker B. M., Dommisch H., Dale B. A., Chung W. O. (2009). Modulation of expression of innate immunity markers CXCL5/ENA-78 and CCL20/MIP3*α* by protease-activated receptors (PARs) in human gingival epithelial cells. *Innate Immunity*.

[8] Uehara A., Muramoto K., Imamura T. (2005). Arginine-specific gingipains from *Porphyromonas gingivalis* stimulate production of hepatocyte growth factor (scatter factor) through protease-activated receptors in human gingival fibroblasts in culture. *The Journal of Immunology*.

[9] Pagel C. N., Song S.-J., Loh L. H. (2009). Thrombin-stimulated growth factor and cytokine expression in osteoblasts is mediated by protease-activated receptor-1 and prostanoids. *Bone*.

[10] Arayatrakoollikit U., Pavasant P., Yongchaitrakul T. (2008). Thrombin induces osteoprotegerin synthesis via phosphatidylinositol 3′-kinase/mammalian target of rapamycin pathway in human periodontal ligament cells. *Journal of Periodontal Research*.

[11] Uehara A., Imamura T., Potempa J., Travis J., Takada H. (2008). Gingipains from *Porphyromonas gingivalis* synergistically induce the production of proinflammatory cytokines through protease-activated receptors with Toll-like receptor and NOD1/2 ligands in human monocytic cells. *Cellular Microbiology*.

[12] Rovai E. S., Holzhausen M. (2017). The role of proteinase-activated receptors 1 and 2 in the regulation of periodontal tissue metabolism and disease. *Journal of Immunology Research*.

[13] Ohuchi N., Hayashi K., Iwamoto K. (2010). Thrombin-stimulated proliferation is mediated by endothelin-1 in cultured rat gingival fibroblasts. *Fundamental & Clinical Pharmacology*.

[14] Yang W. H., Deng Y. T., Hsieh Y. P., Wu K. J., Kuo M. Y. P. (2016). Thrombin activates latent TGF*β*1 via integrin *α*v*β*1 in gingival fibroblasts. *Journal of Dental Research*.

[15] da Silva H. A. B., Alves V. T. E., Spolidório L. C. (2014). Expression of protease activated receptor-1 in chronic periodontitis. *Journal of Periodontology*.

[16] Mackie E., Loh L., Sivagurunathan S. (2008). Protease-activated receptors in the musculoskeletal system. *The International Journal of Biochemistry & Cell Biology*.

[17] Sato N., Ichikawa J., Wako M. (2016). Thrombin induced by the extrinsic pathway and PAR-1 regulated inflammation at the site of fracture repair. *Bone*.

[18] Abraham L. A., Mackie E. J. (1999). Modulation of osteoblast-like cell behavior by activation of protease-activated receptor-1. *Journal of Bone and Mineral Research*.

[19] Song S. J., Pagel C. N., Campbell T. M., Pike R. N., Mackie E. J. (2005). The role of protease-activated receptor-1 in bone healing. *The American Journal of Pathology*.

[20] Kanno Y., Ishisaki A., Kawashita E., Kuretake H., Ikeda K., Matsuo O. (2016). uPA attenuated LPS-induced inflammatory osteoclastogenesis through the plasmin/PAR-1/Ca^2+^/CaMKK/AMPK axis. *International Journal of Biological Sciences*.

[21] Somerman M. J., Archer S. Y., Imm G. R., Foster R. A. (1988). A comparative study of human periodontal ligament cells and gingival fibroblasts in vitro. *Journal of Dental Research*.

[22] Hollenberg M. D., Saifeddine M., al-Ani B., Kawabata A. (1997). Proteinase-activated receptors: structural requirements for activity, receptor cross-reactivity, and receptor selectivity of receptor-activating peptides. *Canadian Journal of Physiology and Pharmacology*.

[23] Maryanoff B. E., Zhang H. C., Andrade-Gordon P., Derian C. K. (2003). Discovery of potent peptide-mimetic antagonists for the human thrombin receptor, protease-activated receptor-1 (PAR-1). *Current Medicinal Chemistry-Cardiovascular & Hematological Agents*.

[24] Gregory C. A., Grady Gunn W., Peister A., Prockop D. J. (2004). An alizarin red-based assay of mineralization by adherent cells in culture: comparison with cetylpyridinium chloride extraction. *Analytical Biochemistry*.

[25] Choi J. K., Hwang H. I., Jang Y. J. (2015). The efficiency of the in vitro osteo/dentinogenic differentiation of human dental pulp cells, periodontal ligament cells and gingival fibroblasts. *International Journal of Molecular Medicine*.

[26] Ozeki N., Mogi M., Hase N. (2016). Polyphosphate-induced matrix metalloproteinase-13 is required for osteoblast-like cell differentiation in human adipose tissue derived mesenchymal stem cells. *Bioscience Trends*.

[27] Lian J. B., Javed A., Zaidi S. K. (2004). Regulatory controls for osteoblast growth and differentiation: role of Runx/Cbfa/AML factors. *Critical Reviews in Eukaryotic Gene Expression*.

[28] Song S. J., Pagel C. N., Pike R. N., Mackie E. J. (2005). Studies on the receptors mediating responses of osteoblasts to thrombin. *The International Journal of Biochemistry & Cell Biology*.

[29] Coughlin S. R. (1999). Protease-activated receptors and platelet function. *Thrombosis and Haemostasis*.

[30] Coughlin S. R. (2000). Thrombin signalling and protease-activated receptors. *Nature*.

[31] Kirilak Y., Pavlos N., Willers C. (2006). Fibrin sealant promotes migration and proliferation of human articular chondrocytes: possible involvement of thrombin and protease-activated receptors. *International Journal of Molecular Medicine*.

[32] Wang H., Ubl J. J., Stricker R., Reiser G. (2002). Thrombin (PAR-1)-induced proliferation in astrocytes via MAPK involves multiple signaling pathways. *American Journal of Physiology-Cell Physiology*.

[33] Xu J., Li Z., Hou Y., Fang W. (2015). Potential mechanisms underlying the Runx2 induced osteogenesis of bone marrow mesenchymal stem cells. *American Journal of Translational Research*.

[34] Ducy P., Zhang R., Geoffroy V., Ridall A. L., Karsenty G. (1997). Osf2/Cbfa1: a transcriptional activator of osteoblast differentiation. *Cell*.

[35] Komori T., Yagi H., Nomura S. (1997). Targeted disruption of *Cbfa1* results in a complete lack of bone formation owing to maturational arrest of osteoblasts. *Cell*.

[36] Lee M. H., Javed A., Kim H. J. (1999). Transient upregulation of CBFA1 in response to bone morphogenetic protein‐2 and transforming growth factor *β*1 in C2C12 myogenic cells coincides with suppression of the myogenic phenotype but is not sufficient for osteoblast differentiation. *Journal of Cellular Biochemistry*.

[37] Ram V. S., Parthiban, Sudhakar U., Mithradas N., Prabhakar R. (2015). Bonebiomarkers in periodontal disease: a review article. *Journal of Clinical and Diagnostic Research*.

